# The future of atrial fibrillation therapy: intervention on heat shock proteins influencing electropathology is the next in line

**DOI:** 10.1007/s12471-015-0699-0

**Published:** 2015-05-07

**Authors:** E.A.H. Lanters, D.M.S. van Marion, H. Steen, N.M.S. de Groot, B.J.J.M. Brundel

**Affiliations:** 1 Unit Translational Electrophysiology, Department of Cardiology, Erasmus Medical Center, Rotterdam, The Netherlands; 2 Department of Clinical Pharmacy and Pharmacology, EB71, University Medical Center Groningen, University of Groningen, Hanzeplein 1, PO Box 30 001, 9700RB Groningen, The Netherlands; 3 Nyken Therapeutics, BV, Groningen, The Netherlands; 4 Thorax Center’s, Gravendijkwal 230, 3015 CE Rotterdam, The Netherlands; 5 Department of Physiology, Institute for Cardiovascular Research, VU University Medical Center, Amsterdam, The Netherlands

**Keywords:** Atrial fibrillation, Mapping, Electropathology, Heat shock proteins, Geranylgeranylacetone

## Abstract

Atrial fibrillation (AF) is the most common age-related cardiac arrhythmia accounting for one-third of hospitalisations. Treatment of AF is difficult, which is rooted in the progressive nature of electrical and structural remodelling, called electropathology, which makes the atria more vulnerable for AF. Importantly, structural damage of the myocardium is already present when AF is diagnosed for the first time. Currently, no effective therapy is known that can resolve this damage.

Previously, we observed that exhaustion of cardioprotective heat shock proteins (HSPs) contributes to structural damage in AF patients. Also, boosting of HSPs, by the heat shock factor-1 activator geranylgeranylacetone, halted AF initiation and progression in experimental cardiomyocyte and dog models for AF. However, it is still unclear whether induction of HSPs also prolongs the arrhythmia-free interval after, for example, cardioversion of AF.

In this review, we discuss the role of HSPs in the pathophysiology of AF and give an outline of the HALT&REVERSE project, initiated by the HALT&REVERSE Consortium and the AF Innovation Platform. This project will elucidate whether HSPs (1) reverse cardiomyocyte electropathology and thereby halt AF initiation and progression and (2) represent novel biomarkers that predict the outcome of AF conversion and/or occurrence of post-surgery AF.

## Introduction

Atrial fibrillation (AF) represents the arrhythmia with the highest prevalence, accounting for one-third of hospitalisations related to cardiac rhythm disturbances [1]. The main risk factors to develop AF include age, cardiac surgery, valvular heart disease, congestive heart disease, ischaemic cardiomyopathy, obesity, hypertension and diabetes mellitus. These risk factors cause atrial stretch and dilation and condition the heart for AF [1]. Patients with AF are frequently encountered in daily clinical practice. The goal of therapy in AF is, ideally, to abolish AF episodes and to restore sinus rhythm. This in turn re-establishes atrioventricular synchrony and improves atrial function. Despite extensive research, treatment of AF remains difficult, which is rooted in the persistent and progressive nature of this arrhythmia. An initiator, trigger and substrate are mandatory for development of AF. In this manuscript, we will focus on the substrate underlying AF.

AF persistence is caused by progressive changes in the electrophysiology and structure of atrial cardiomyocytes [2], as demonstrated in a goat model for AF. In this model, AF resulted in shortening of the atrial effective refractory period and reversion of the physiological rate adaptation (shortening of the atrial refractory period at slower heart rates), facilitating induction and stability of AF. Similar findings have been reported in humans. The goat model of AF also revealed that longstanding AF is associated with changes in myocardial structure, including dedifferentiation, increase in cell size, perinuclear accumulation of glycogen, central loss of sarcomeres, changes in mitochondrial shape, fragmentation of the sarcoplasmatic reticulum and disorganisation of fibre orientation [3]. These structural changes were observed in human AF as well [4]. Importantly, structural changes are sustainable and impair electrical coupling and the functional recovery to sinus rhythm by pharmacological and electrical cardioversion. In addition, it was found that alterations of the myocardial structure underlie changes in electrophysiology as observed in AF [2, 5]. This phenomenon is commonly defined as electropathology. It should be noted that electropathology is already present when a patient enters the clinic for the first time with an episode of AF. The currently available pharmacological therapy is directed at alleviation of electrical changes (rhythm control) and has limited effect on patient outcome [1]. Therapeutic approaches that halt the mechanisms conveying the AF-induced structural remodelling may offer superior therapeutic perspectives. Thus, from science to patients: reversal of electropathology of cardiomyocytes represents a key target to accomplish and maintain cardiac sinus rhythm after AF.

## Discontinuous propagation in the atria

**Fig. 1:**
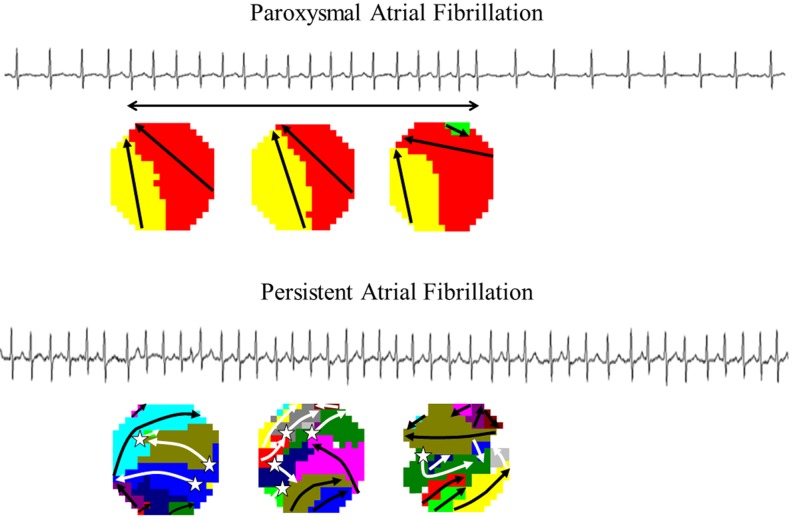
Persistence of atrial fibrillation. Surface electrograms of paroxysms (*upper panel*) and persistent (*lower panel*) atrial fibrillation. Wave-maps constructed during these different types of maps are also shown. A wave-map shows individual fibrillation waves represented by colours according to their sequence of appearance. A previously described mapping algorithm was used to classify them into peripheral waves (entering the mapping area from outside the electrode array), breakthrough waves (appearing at the epicardial surface inside the mapping area) or discontinuous conduction waves (fibrillation waves starting with a delay of 13–40 ms from boundaries of other waves). Sites of epicardial breakthroughs are indicated by *white asterisks*; *white arrows* indicate direction(s) of expansion of epicardial breakthrough or discontinuous fibrillation waves. Peripheral fibrillation waves are indicated by *black arrows*

**Fig. 2:**
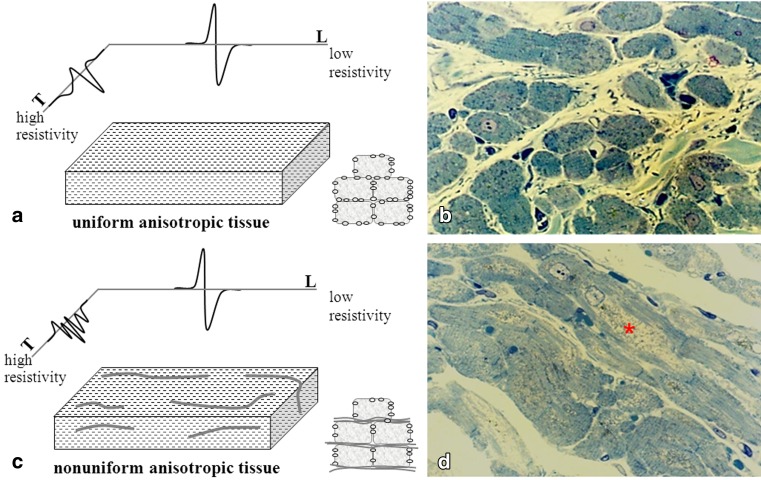
Discontinuous conduction. **a** Electrograms recorded in the longitudinal and transversal conduction direction in relation to fibre orientation in uniform anisotropic and non-uniform anisotropic tissue. The electrogram in the transverse conduction direction in non-uniform anisotropic tissue consists of multiple deflections indicating discontinuous conduction. These alterations may be caused by changes in myocardial structure, including increase in cell size, perinuclear accumulation of glycogen, central loss of sarcomeres (myolysis), changes in mitochondrial shape, fragmentation of the sarcoplasmatic reticulum and disorganisation of fibre orientation as (partly) observed in panel **b** and **c**. **b** Atrial myocardium from a patient in sinus rhythm (SR) and (**c**) a patient with permanent AF (PeAF) stained with toluidine blue. SR patients show normal structural cardiomyocytes, whereas the patient with PeAF reveals cardiomyocytes with extensive perinuclear myolysis (*red asterisk*)

It is generally assumed that transition of short-lasting paroxysms of AF to long-lasting episodes that no longer terminate spontaneously over time corresponds to a progression from a trigger-driven to a substrate-mediated arrhythmia due to a combination of electrical, structural and contractile atrial remodelling (Fig. 1). Previous studies have demonstrated that alterations in the atrial microscopic structure impair intra-atrial conduction [6]. Spach et al. [7, 8] were the first to provide evidence that *two-dimensional propagation is discontinuous in nature at a microscopic level* (Fig. 2). In their experiments, variations in shape and amplitude of extracellular potential waveforms were studied when the direction of conduction was changed from longitudinal to transverse in relation to the fibre axis. They observed that in uniform anisotropic myocardium, the shape of the upstroke of the transmembrane action potential depended on the direction of propagation in relation to the fibre orientation. Fast conduction in the longitudinal direction was associated with a slow upstroke and a long τ-foot (time constant of the action potential representing voltage decay) and slow conduction in the transverse direction with a fast upstroke and a short τ-foot. Differences were explained by the anisotropic distribution of intercellular connections (gap junctions, connexions). This was confirmed in a goat model for sustained (2 months) AF, where persistence of AF was associated with a lower density and inhomogeneous distribution of gap junctions [9, 10]. Gap junctions along the fibre axis provide a low resistance to current flow that increases the space constant and hence the electrotonic current flow. Subsequently, the upstroke of the transmembrane potential in this direction decreases and the τ-foot increases. Due to the high resistance barriers in the transverse direction, the space constant is reduced and the electrotonic current is therefore smaller. This results in an increase in the upstroke of the transmembrane potential and a decrease in the τ-foot. Consequently, as the safety factor is lower during longitudinal conduction than during transverse conduction, conduction block is more likely to occur during longitudinal conduction [11].

Next to gap junctional remodelling, also ion channel remodelling has been demonstrated to play a role in AF progression. A marked reduction in ion (preferably L-type Ca^2+^ and transient outward K^+^) channel protein expression, associated with a shortening of atrial effective refractory period, was observed in patients with AF [12]. This decrease in protein expression might be explained by increased (calcium dependent) calpain I activity, causing enhanced degradation of ion-channel proteins and contractile proteins in the myocardium [4, 13].

Modifications of the atrial architecture affecting cardiomyocyte geometry (size and shape), gap junctions (distribution and conductivity) or the interstitial space (size and distribution) result in non-uniform anisotropic tissue. Muscle bundles become electrically dissociated due to strands of fibrotic tissue giving rise to fractionation of extracellular potentials [14]. These areas of local conduction abnormalities may facilitate genesis and perpetuation of AF.

## Fractionation of extracellular potentials

Fractionated electrograms are extracellular waveforms containing multiple, distinct deflections reflecting local asynchronous activation of myocardium surrounding the recording electrode [14]. Fractionation of fibrillation potentials can be functional and/or structural in nature as local asynchronous activation can be the result of a spatial dispersion in refractory periods (functional) or non-uniform tissue anisotropy (structural, as explained above). During AF, multiple, simultaneously circulating wavelets excite the atria. A fibrillation wave may encounter atrial tissue that is partially or totally refractory due to previous excitation by another wave. This results in slowing of conduction, conduction block and turning of fibrillation waves around lines of conduction block. All these conduction abnormalities give rise to local asynchronous activation of the myocardium and thus to multiple deflections in the extracellular potentials.

**Fig. 3:**
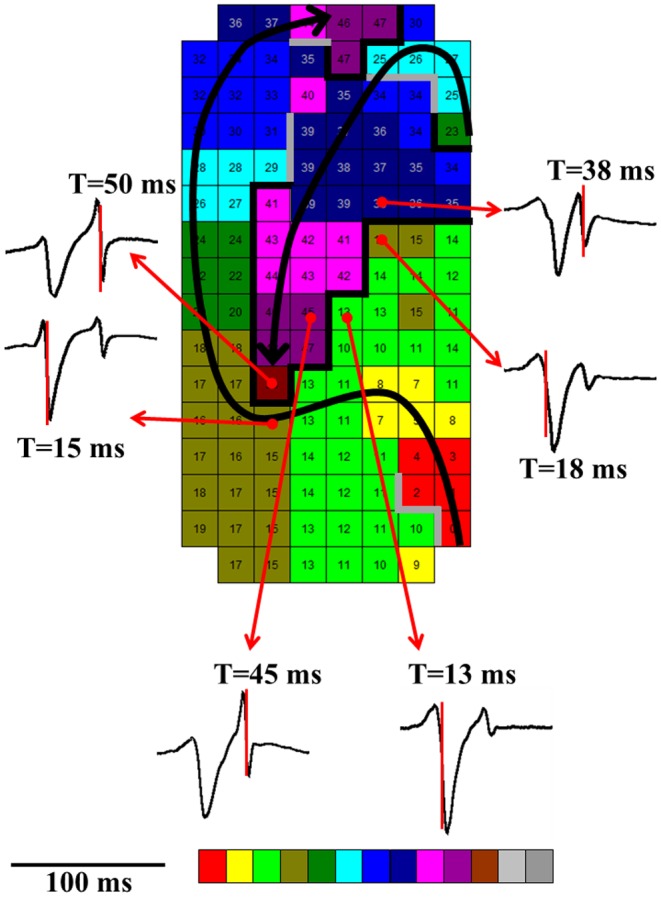
Double potentials recorded along the lines of conduction block. The colour-coded activation map (interelectrode distance, 2 mm) shows two fibrillation waves (its trajectories indicated by *thick black lines*) separated by areas of conduction block. Electrograms consisting of two deflections are shown outside the activation map. They are recorded along the line of conduction block reflecting activation on either side

High-density epicardial mapping during electrically induced AF in humans without structural heart disease showed that there is a relation between the degree of fractionation in the right atrium and specific spatial patterns of activation. Double potentials were recorded along the lines of conduction block (Fig. 3), whereas electrograms with three or more deflections were recorded at pivot points or areas of slow conduction [15, 16]. In older patients with structural heart disease and longstanding persistent AF, it is likely that fractionation is more structurally determined due to dissociation of muscle bundles by fibrosis or fatty degeneration. Recent studies suggested that ablation of areas with fractionated electrograms may eliminate AF suggesting that fractionated electrograms are indeed indicative for arrhythmogenic regions perpetuating AF [17].

## Counteracting electropathology by heat shock proteins

Previous research has unequivocally demonstrated that the induction of the heat shock response provides protection against various cardiac diseases [18]. Sensing of cellular stress activates the heat shock transcription factor-1 (HSF-1) and consequently results in the expression of heat shock proteins (HSPs). In general, HSPs function as intra-cellular chaperones for other proteins. They play an important role in protein–protein interactions such as folding and assisting in the establishment of proper protein conformation (shape) and prevention of unwanted protein aggregation, which occur during ageing of the cell and (cardiac) stress [18]. Because of their essential role in protein maintenance, some members of the HSP family are expressed at low to moderate levels in all organisms. In cardiomyocytes, HSPs bind to damaged proteins, including sarcomeric proteins, and thereby prevent cardiomyocyte toxicity and further structural remodelling and functional loss [19, 20]. HSPs consist of five HSP families, including the HSP70 and HSP27 family, which have both been suggested to convey cardioprotective effects [19]. Therefore, impairment of HSP expression, especially HSP70 and HSP27, has been related to the development of various cardiac diseases, including AF [20], but also metabolic diseases, such as diabetes and obesity [21], which trigger the induction of AF [1].

These findings indicate that HSPs represent an interesting target to treat cardiac diseases such as AF. Indeed, experimental and human studies revealed that HSPs provide protection against AF initiation. In dogs, pretreatment with the HSP inducer, geranylgeranylacetone (GGA), attenuates changes in electrophysiology and conduction velocity of atrial cardiomyocytes and prevents initiation of AF in (acute) ischaemia [22]. Also protective effects of HSPs were observed in a rabbit model for AF induced by heart failure [23]. A further indication for the protective effect of HSPs is obtained from two studies in patients undergoing cardiac surgery. In both studies an inverse correlation between higher levels of HSP70 expression in atrial tissue and lower incidence of post-operative AF was observed [24, 25]. In addition, one study described a correlation between HSP70 and HSP27 levels in tissue and improved restoration of sinus rhythm after mitral valve surgery [26]. In addition, high HSP27 levels in blood predict sinus rhythm maintenance after catheter ablation in patients with paroxysmal AF [26]. In an experimental study in mice, it was observed that induction of HSP70 prevents angiotensin II-induced AF and limits atrial fibrosis formation [27]. Together, these results indicate that induction of HSPs mitigates the development of the atrial substrate for the induction of AF.

In addition to the cardioprotective effect of HSPs against the induction of AF, it is expected that induced expression of HSPs also protects against AF progression. Such a cardioprotective effect is supported by several studies. For example, general HSP levels were induced by a heat shock or GGA treatment in tachypaced HL-1 atrial cardiomyoyctes and these cardiomyocytes were compared with normal tachypaced HL-1 cardiomyocytes [20, 28]. In these studies, tachypaced cardiomyocytes with induced HSP expression revealed improved contractile function and structural integrity. Similar protection by general HSP induction was obtained in a dog model for AF [28]. Dogs pretreated with GGA showed attenuation of tachypacing-induced AF promotion and limited recurrence of AF after cardioversion, most likely by limiting the electrophysiological changes, including shortening of action potential duration and maintenance of L-type Ca^2+^ current [28]. Further studies revealed that specific members of the family of small HSPs play a key role in cardioprotection against AF. Induction of HSP27 was found sufficient to prevent tachypacing-induced structural remodelling, while overexpressing HSP70 was not protective [28]. Other specific small HSP family members also show protection in cellular models, including HSP20, cvHSP and HSP22, but not the other small HSP family members [29]. Importantly, selective knockdown of HSP27 fully attenuates the protective effect of GGA, demonstrating a main role for HSP27 in cardioprotection. A similar protective effect of the small HSP members as observed in HL-1 cardiomyocytes has been found in the *Drosophila melanogaster* (fruit fly) model for tachypacing-induced contractile dysfunction. In the transparent fruit fly pupae, the heart wall can be visualised and subjected to tachypacing [30]. This model also allows for general induction of HSPs, either by heat shock or by HSP-inducing compounds such as GGA, or specific induction of HSPs via genetic manipulation specifically in the heart. Indeed, induction of HSPs, especially the small HSP, by these strategies prevents tachypacing-induced contractile dysfunction and structural remodelling in *Drosophila* pupae [30]. Also in human AF, there are indications that HSP27 may protect against cardiac remodelling and AF progression. Two independent studies report higher atrial expression levels of HSP27 to relate to a shorter duration of AF and less extensive structural damage [20, 31]. This suggests that in short-duration AF, the HSP response gets activated, while it diminishes over time when AF persists. As HSP27 conserves the cardiomyocyte structure, lower levels of HSP27 may trigger the progression of structural remodelling paving the way to longstanding and permanent AF. Therefore, securing HSP levels at an adequate level, for example, by treatment with HSP inducers, may limit the expansion of the AF substrate during paroxysmal and short-term AF. In agreement with this hypothesis, restoration of sinus rhythm in patients with permanent AF after mitral valve surgery is related to the heat shock factor (HSF) -1 activity and induced HSP27 levels [32].

Taken together, induction of HSPs attenuates electropathology of cardiomyocytes in AF. In Hsf1 Activators Lower cardiomyocyte damage: Towards a novel therapeutic approach to REVERSE atrial fibrillation (HALT & REVERSE), we will focus on the electropathological substrate of AF and dissect the mechanism of protection.

## Novel HSP-inducing agents as a therapeutic approach in AF

Pharmacological approaches to prevent mechanisms underlying atrial electropathology are being studied, with the hope that they might be useful therapeutic agents in treating AF [1]. It has been recognised that the efficacy of commonly used drugs on remodelling is limited [1]. As the protective action of HSPs depends on their temporary induction, drugs that boost the endogenous heat shock responses are of particular interest in the prevention of first onset, recurrence and progression of AF. A drug often used to boost HSP expression is GGA. GGA was originally used as an anti-ulcer agent and is a non-toxic acyclic isoprenoid compound with a retinoid skeleton that induces HSP synthesis in various tissues, including gastric mucosa, intestine, liver, heart, retina and the central nervous system [33, 34]. GGA induces HSP expression through the activation of HSF-1. The protective effect of GGA-induced HSP expression on electropathology caused by tachycardia has been observed in various experimental models, including the dog model for AF [28]. These findings suggest that the induction of HSPs by GGA might have a potential value for clinical AF. In in vivo tachypaced *Drosophila*, GGA treatment protected against contractile dysfunction of the heart wall and structural remodelling [30]. Also in the dog model for (acute) atrial ischaemia and tachypacing-induced AF promotion, an HSP-inducing GGA treatment revealed protective effects against cardiomyocyte remodelling, and consequently occurrence and recurrence of AF [22]. A direct protective effect of HSP on the earlier-mentioned gap junctional and ion channel remodelling has not been described (yet). However, it has been shown that GGA treatment attenuates the proteolytic activity of calpain [30] and prevents ion channel remodelling in AF [28], suggesting a direct protective role of HSPs.

**Fig. 4:**
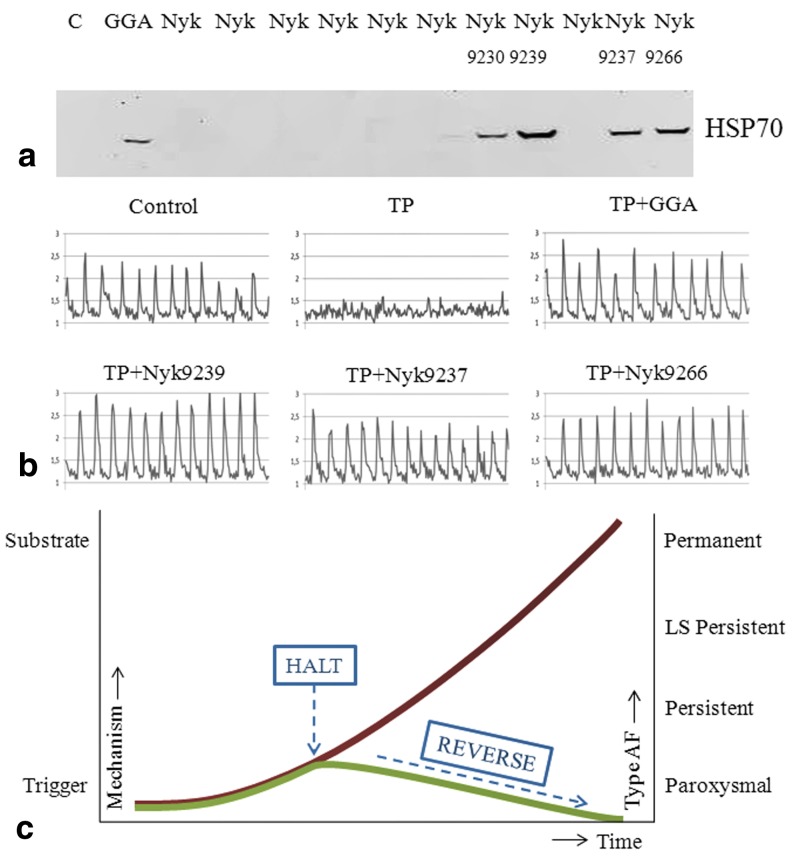
Geranylgeranylacetone (GGA) derivatives induce HSP70 expression and reveal cardioprotective effects. **a** Western blot showing induction of HSP70 levels in HL-1 cardiomyocytes treated with GGA and some of the GGA derivatives (Nyk compounds). **b** Representative calcium transient tracers of non-treated and normally paced (1 Hz) HL-1 cardiomyocytes (*Control*) or tachypaced cardiomyocytes (*TP*, 5 Hz) without or with treatment of 10 µM GGA or GGA derivatives (Nyk compounds) as indicated. **c** Overview of the central concept of HALT&REVERSE. Atrial fibrillation (AF) naturally progresses in time (*red line*), which is rooted in the underlying electropathology. By pharmacological induction of HSP levels (*blue arrow*), we aim to halt or even reverse electropathology and consequently AF progression (*green line*)

Although protective effects with GGA were observed, an important disadvantage of GGA is its high LogP value, which is around 9, and therefore generally high dosages are required, as found in the dog studies for AF [22, 28]. To overcome this disadvantage, various derivatives of GGA have been synthesised, with improved pharmaco-chemical and HSP-boosting properties (Fig. 4a). Some of these derivatives also showed cardioprotective effects in tachypaced HL-1 cardiomyocytes (Fig. 4b). In the HALT&REVERSE project, we will test whether these derivatives reveal improved cardioprotective effects compared with GGA and elucidate their mode of action. In addition, one of the selected GGA derivatives will be further tested in an animal model for AF. Finally, GGA has also been tested in patients undergoing cardiac surgery, and atrial tissue will be used to test whether GGA induces HSP expression in patients and protects against post-surgery AF.

## Expected findings of HALT&REVERSE

Ultimately, by inducing cardioprotective HSP expression, the HALT&REVERSE Consortium wants to halt or even reverse the structure of cardiomyocytes and therefore the electropathology in patients with AF (Fig. 4c). Hereby, we expect to significantly contribute to novel rational approaches, to enhance successful recovery of heart function after electrical cardioversion (ECV) and/or cardiac surgery. This approach will affect the substrate underlying AF, not the trigger. For the first time, we exploit HSP levels to predict the electropathological outcome in patients, as measured by our unique mapping approaches. In addition, at the end of this project, we will know whether HSPs are suitable biomarkers for AF. Our unique approach will ultimately result in reduced hospital time, induced quality of life for the patient and lower costs for society. The findings will be of benefit to other (cardiac, kidney) diseases, as many of those are characterised by sustainable structural remodelling. Thus, the project is of major importance from a scientific, clinical and economical perspective.
